# Supplementary material for: Markers of Angiogenesis, Lymphangiogenesis, and Epithelial–Mesenchymal Transition (Plasticity) in CIN and Early Invasive Carcinoma of the Cervix: Exploring Putative Molecular Mechanisms Involved in Early Tumor Invasion
Source: Int J Mol Sci. 2020 Sep 6;21(18):6515. doi: 10.3390/ijms21186515 (PMC7554870; doi:10.3390/ijms21186515)
Supplement: Supplementary file 1 [file ijms-21-06515-s001.zip › Supplementary Files - revised/Table S1.docx]

**Table S1.** The list of genes selected to characterize the expression profiles of markers of epithelial (group A) or mesenchymal (group B) tissue organization, (lymph-)angiogenesis and epithelial-mesenchymal trans-differentiation (group C) in the original RNA-Seq dataset (supplemental to Figures 2 and 4).

| **GROUP A**: EPITHELIAL phenotype markers | | |
| --- | --- | --- |
| CYTOSKELETON | | CGN, CGNL1, CNFN, CRNN, CSTA, CSTB, DSP, EVPL, FLG, JUP, KAZN, KRT1, KRT2, KRT4, KRT6A, KRT6B, KRT6C, KRT9, KRT10, KRT13, KRT16, MLLT4, PKP1, PKP2, PKP3, PPL, SCEL, ZO-1/TJP1 |
| ADAPTOR PROTEINS, SIGNAL TRANSDUCERS | | ARF6, CALML3, CRABP2, DMKN, FABP5, KANK1, KRTDAP, S100A10, S100A11, SFN, TMOD3 |
| ADHESION MOLECULES, EXTRACELLULAR MATRIX COMPONENTS | | CDH1, DSC2, DSG1, DSG2, DSG3, EMP1, EPCAM, IVL, LCE3A, LCE3D, LCE3E, MPP7, OCLN, PERP, RPTN, SBSN, SPRR1A, SPRR1B, SPRR2A, SPRR2D, SPRR2E, SPRR3 |
| EXTRACELLULAR MATRIX REMODELING ENZYMES | | CERS3, DESC1, KLK5, KLK6, KLK7, KLK8, KLK12, KLK13, PI3, PPP1R3C, SERPINB3, SERPINB4, SERPINB5, SERPINB13, SPINK5, TGM1, TGM2, TGM3 |
| **GROUP B**: MESENCHYMAL phenotype markers | | |
| CYTOSKELETON | | ACTN1, aSMA/ACTA2, DST, EZR, FLNA, FLNB, FSCN1, KRT5, KRT8, KRT14, KRT15, KRT17, KRT18, KRT19, MACF1, MSN, PALLD, PLEK2, TAGLN, TLN1, TNS4, VCL, VIM, ZYX |
| ADHESION MOLECULES, EXTRACELLULAR MATRIX COMPONENTS | | BCAM, CD44, CD133, CDH2, CDH3, COL1A1, COL1A2, DDR1, FAT1, FAT2, FBLN1, FN1, ITGA2, ITGA3, ITGA5, ITGA6, ITGB1, ITGB4, LAMA3, LAMB1, LAMB2, LAMB3, LAMC2, LUM, MUC5B, MUC6, MUC16, TNC |
| EXTRACELLULAR MATRIX REMODELING ENZYMES | | MMP2, MMP7, MMP9, MMP12, MMP14, SERPINB1, t-PA/PLAT, u-PA/PLAU |
| **GROUP C**: ANGIOGENESIS , LYMPHANGIOGENESIS, EPITHELIAL-TO-MESENCHYMAL TRANSITION | | |
| **(LYMPH-)ANGIOGENESIS :**  **Positive regulators** | GROWTH FACTORS, CYTOKINES, CHEMOKINES | ANG, ANGPT2, CCL2, CCL19, CCL20, CCL21, CCL28, CSF1, CTGF, CXCL1, CXCL3, CXCL5, CXCL12/SDF1, CXCL13, CXCL17, ECSCR, EDN1, EDN2, EREG, IGF1, IL7, IL8, MDK, PDGFA, PDGFB, PDGFC, PDGFD, PGF, PTN, TIMP1, VEGFA, VEGFB, VEGFC, VEGFD |
|  | RECEPTORS for GROWTH FACTORS, CYTOKINES, CHEMOKINES | CCR7, CSF1R, CXCR2, CXCR4, ECE1, EGFR, EFNA1, EFNB1, ENG, EPHA2, EPHB2, EPHB4, FGFR2, FGFR3, GPC1, IL17RB, MCAM, NRP1, NRP2, PDGFRA, PDGFRB, PLAUR, PLXNA1, PLXNB1, PLXNB2, PLXND1, RYK, SEMA4A, SEMA4B, SEMA4C, SEMA6A, TIE1, VEGF-R1/FLT1, VEGF-R3/FLT4 |
|  | ADHESION MOLECULES, ECM COMPONENTS | CD34, ECM1, ESAM, FMOD, GJA1, GJB2, ITGA1, ITGA2, ITGA4, ITGA9, ITGAV, ITGB1, ITGB2, LGALS1, LGALS3, LGALS8, LYVE1, MCAM, MUC1, MUC4, PDPN, PECAM1, PODXL, POSTN, PXN, SRGN, STAB1, TFF3, TGFBI, VCAM1, VCAN, VWF |
|  | TRANSCRIPTION FACTORS | EDF1, ELF2, ELF3, ELF4, ERG, ETS1, ETS2, HIF1A, HIF2A/EPAS1, NFATC1, PROX1, SOX18, THAP1, TTF1, VEZF1 |
|  | ADAPTOR PROTEINS, SIGNAL TRANSDUCERS | ADAM8, AKT3, BCAS3, FGL2, S100A8, S100A9, TSPAN1, TSPAN3, TYMP |
| **EPITHELIAL-to-MESENCHYMAL TRANSITION: Positive regulators** | GROWTH FACTORS, CYTOKINES | HGF, IL1B, IL6, SPP1, TGFB1, WISP1, WNT2B, WNT5A |
|  | RECEPTORS for GROWTH FACTORS, CYTOKINES | B7-H3/CD276, FZD6, JAG1, LRP5, MET, NOTCH1, NOTCH2, NOTCH3, OSMR, PLAUR, RYK, SMO, TGFBR1, TGFBR2 |
|  | TRANSCRIPTION FACTORS | BNC1, FOS, FOXC1, FOXC2, FOXM1, FOXO1, FOXO3, FOXQ1, HMGB1, HMGB2, NANOG, OCT4, SLUG/SNAI2, SNAI1, TEAD2, TEAD4, TWIST1, TWIST2, ZEB1, ZEB2, YBX-1 |
|  | ADAPTOR PROTEINS, SIGNAL TRANSDUCERS | ANXA2, ANXA5, ANXA7, CTNNB1, DVL3, ILK, MEMO1, MIEN1, PAK4, RHOB, RHOC, S100A2, S100A4, S100A6, S100A7, S100A8, S100A9, SMAD2, SMAD3, SMAD4, STAT3, STMN1, TRIO, TRIP6, TRIP10, USP22 |
| **(LYMPH-) ANGIOGENESIS : Negative regulators** | | ALK1, BMP9, CCBE1, COL18A1, DAAM1, DCN, EFNA5, EMP1, SEMA3A, SEMA3C, SEMA3D, SEMA3E, THBS1, THBS2, THBS3, VASH1 |
| **EPITHELIAL-to-MESENCHYMAL TRANSITION : Negative regulators** | | ANXA1, BARX2, CRYAB, EHF, ESR1, GRHL1, GRHL3, HOPX, KLF4, KLF5, KLF8, TRPS1, S100A14, S100A16, SPINK5, SPINK7 |
