# Supplementary material for: Markers of Angiogenesis, Lymphangiogenesis, and Epithelial–Mesenchymal Transition (Plasticity) in CIN and Early Invasive Carcinoma of the Cervix: Exploring Putative Molecular Mechanisms Involved in Early Tumor Invasion
Source: Int J Mol Sci. 2020 Sep 6;21(18):6515. doi: 10.3390/ijms21186515 (PMC7554870; doi:10.3390/ijms21186515)
Supplement: Supplementary file 1 [file ijms-21-06515-s001.zip › Supplementary Files - revised/Table S4.docx]

**Table S4.** Enriched GO Biological Process terms for the 201 genes differentially expressed between early invasive CR and CIN. The GO functions are arranged in ascending order according to the FDR value (supplemental to Figure 10).

| **Enrichment FDR** | **Genes in list** | **Total genes** | **Functional Category** | **Genes** |
| --- | --- | --- | --- | --- |
| 1,99E-08 | 35 | 1062 | Tube development | ANXA1 DCN SLIT2 IL18 CXCL10 EPGN CX3CL1 TYMP PGR APOB PCSK5 MTHFD1 CRISPLD2 AGR2 ALOX12 EREG EPHB2 BRIP1 SHROOM3 PML EDNRA HPGD GJB2 E2F7 ANPEP AR NR2F2 PTCH1 FAT4 MME SASH1 PTPRM CD24 TGFBR3 SPINK5 |
| 4,38E-07 | 29 | 860 | Tube morphogenesis | ANXA1 DCN SLIT2 IL18 CXCL10 EPGN CX3CL1 TYMP PGR APOB MTHFD1 AGR2 ALOX12 EREG EPHB2 SHROOM3 PML EDNRA HPGD E2F7 ANPEP AR NR2F2 PTCH1 FAT4 SASH1 PTPRM TGFBR3 SPINK5 |
| 2,51E-06 | 30 | 1006 | Response to lipid | ANXA1 IL1RN CXCL9 CXCL10 CFTR CX3CL1 TGFBR3 IL18 AR DCN PTPRU ADCYAP1R1 PGR APOB DKK1 ALOX12 CRYAB SLPI DSG1 PAM LCN2 HMGB2 HPGD GJB2 NR2F2 PTCH1 SLIT2 SLIT3 EPHA3 SASH1 |
| 7,09E-06 | 26 | 831 | Epithelial cell differentiation | ANXA1 SPRR3 SPRR2A TGM1 UPK1A SFRP4 CSTA SPINK5 SCEL PGR AGR2 PPL KRT17 SHROOM3 E2F7 AR PTCH1 FAT4 NR2F2 CD24 DHRS9 RHCG EREG DSG1 KLK13 KRT13 |
| 7,09E-06 | 30 | 1077 | Circulatory system development | ANXA1 DCN TGFBR3 DKK1 IL18 CXCL10 EPGN CX3CL1 TYMP APOB PCSK5 MTHFD1 NSD2 EREG EPHB2 PML PAM EDNRA HPGD E2F7 ANPEP SLIT3 NR2F2 PTCH1 FAT4 SASH1 DNMT1 SLIT2 PTPRM SPINK5 |
| 9,19E-06 | 50 | 2561 | Response to external stimulus | CX3CL1 EPHA3 LYZ EPHB2 SAA2 ANXA1 IL1RN CXCL9 SLIT2 KLK7 CXCL10 IL18 OAS3 SLPI EDN3 LCN2 HMGB2 PTPRM SLIT3 SLC7A2 DCN TYMP ACSL4 APOB ALOX12 OAS2 IL1R1 KLK8 SPINK5 NT5E BRIP1 BMPR1B PML NLRC5 ENPP3 HPGD GJB2 UNC5C UPP1 PTCH1 RELN IRF9 STMN1 PPL SCEL SASH1 KIAA1324 SHROOM3 CD24 MMP3 |
| 1,59E-05 | 52 | 2785 | Anatomical structure morphogenesis | EPHA3 KLK8 EPHB2 ANXA1 SLIT2 CDH12 DCN TGFBR3 DKK1 IL1RN IL18 CXCL10 HOPX PTPRM EPGN SLIT3 CX3CL1 TYMP XRCC1 PGR APOB PCSK5 MTHFD1 SFRP4 AGR2 ALOX12 NSD2 CRYAB STMN1 EREG KRT17 BMPR1B CXCL9 SHROOM3 GCNT3 PML PAM EDNRA PPP1R9A HPGD E2F7 ANPEP AR UNC5C NR2F2 PTCH1 RELN FAT4 CRISPLD2 SASH1 LIG1 SPINK5 |
| 1,59E-05 | 44 | 2168 | Tissue development | ANXA1 SPRR3 SPRR2A TGFBR3 TGM1 UPK1A SFRP4 DKK1 CSTA SPINK5 SCEL SLIT2 CXCL10 UNC5C DCN PGR MTHFD1 AGR2 ALOX12 PPL EDN3 HIVEP3 KRT17 BMPR1B SHROOM3 GCNT3 PML EDNRA GJB2 E2F7 AR NR2F2 PTCH1 FAT4 CD24 EPHA3 DHRS9 RHCG EREG EMP1 DSG1 KLK13 KLK7 KRT13 |
| 1,59E-05 | 23 | 715 | Vasculature development | ANXA1 DCN IL18 CXCL10 EPGN CX3CL1 TYMP APOB PCSK5 EREG EPHB2 PML EDNRA HPGD E2F7 ANPEP NR2F2 SASH1 DNMT1 SLIT2 PTPRM TGFBR3 SPINK5 |
| 1,59E-05 | 21 | 603 | Blood vessel morphogenesis | ANXA1 DCN IL18 CXCL10 EPGN CX3CL1 TYMP APOB EREG EPHB2 PML EDNRA HPGD E2F7 ANPEP NR2F2 SASH1 SLIT2 PTPRM TGFBR3 SPINK5 |
| 1,75E-05 | 23 | 724 | Cardiovascular system development | ANXA1 DCN IL18 CXCL10 EPGN CX3CL1 TYMP APOB PCSK5 EREG EPHB2 PML EDNRA HPGD E2F7 ANPEP NR2F2 SASH1 DNMT1 SLIT2 PTPRM TGFBR3 SPINK5 |
| 2,20E-05 | 33 | 1386 | Epithelium development | ANXA1 SPRR3 SPRR2A TGM1 UPK1A SFRP4 CSTA SPINK5 SCEL SLIT2 CXCL10 PGR MTHFD1 AGR2 DKK1 ALOX12 PPL KRT17 SHROOM3 PML EDNRA E2F7 AR PTCH1 FAT4 NR2F2 CD24 DHRS9 RHCG EREG DSG1 KLK13 KRT13 |
| 2,33E-05 | 10 | 125 | Cornification | TGM1 PPL CSTA KRT17 SPINK5 DSG1 SPRR3 KLK13 KRT13 SPRR2A |
| 2,33E-05 | 22 | 687 | Blood vessel development | ANXA1 DCN IL18 CXCL10 EPGN CX3CL1 TYMP APOB PCSK5 EREG EPHB2 PML EDNRA HPGD E2F7 ANPEP NR2F2 SASH1 SLIT2 PTPRM TGFBR3 SPINK5 |
| 9,46E-05 | 36 | 1725 | Response to oxygen-containing compound | ANXA1 IL1RN CXCL9 CXCL10 CFTR CX3CL1 TGFBR3 XRCC1 RDH12 SLIT2 IL18 DCN ADCYAP1R1 APOB DKK1 CRYAB SLPI EREG DNMT1 DSG1 BRIP1 PAM LCN2 HMGB2 HPGD GJB2 AR NR2F2 PTCH1 SLIT3 EPHA3 SASH1 GPR68 GYS2 MMP3 GPX3 |
| 9,46E-05 | 18 | 521 | Epidermis development | ANXA1 SPRR3 SPRR2A TGM1 SFRP4 CSTA SPINK5 SCEL DKK1 PPL KRT17 PTCH1 EREG EMP1 DSG1 KLK13 KLK7 KRT13 |
| 9,46E-05 | 58 | 3547 | Response to organic substance | CX3CL1 ANXA1 IL1RN BMPR1B CXCL9 NLRC5 CXCL10 YOD1 CFTR DCN TGFBR3 AGR2 IL1R1 EREG CILP RDH12 PML SLIT2 TNFRSF21 IL18 HPGD AR CIITA MME PTPRU ACSL4 XRCC1 ADCYAP1R1 PGR APOB MYBL2 SFRP4 DKK1 ALOX12 CRYAB OAS2 SLPI DNMT1 DSG1 BRIP1 PAM LCN2 HMGB2 GJB2 NR2F2 PTCH1 FAT4 SLIT3 EPHA3 LAMP3 SASH1 GPR68 GYS2 CD24 OAS3 MMP3 GPX3 IRF9 |
| 0,00016602 | 41 | 2165 | Cell proliferation | TACC3 EREG ANXA1 TNFRSF21 EPGN PTPRU POLA1 CDKN2C EDN3 CXCL9 IL18 BNIPL HMGB2 AR CXCL10 NCCRP1 DPP4 CD24 PGR ALOX12 KLK8 DNMT1 BRIP1 PML ENPP3 HPGD E2F7 SLIT3 PTCH1 FAT4 CX3CL1 TGM1 SFRP4 PTPRM NR2F2 UHRF1 TGFBR3 BMPR1B EDNRA MXD1 EMP1 |
| 0,0002189 | 11 | 206 | Female pregnancy | HPGD ACSL4 PGR PCSK5 DSG1 PAM GJB2 AR NR2F2 ENDOU CLIC5 |
| 0,00025262 | 17 | 511 | Angiogenesis | ANXA1 DCN IL18 CXCL10 EPGN CX3CL1 TYMP EREG EPHB2 PML EDNRA E2F7 ANPEP SASH1 SLIT2 PTPRM SPINK5 |
| 0,00025262 | 15 | 410 | Epidermal cell differentiation | ANXA1 SPRR3 SPRR2A TGM1 SFRP4 CSTA SPINK5 SCEL PPL KRT17 PTCH1 EREG DSG1 KLK13 KRT13 |
| 0,00025262 | 6 | 47 | Ovulation cycle process | PGR EREG BMPR1B PAM SLIT2 SLIT3 |
| 0,00025262 | 9 | 137 | Response to estradiol | ANXA1 ADCYAP1R1 APOB CRYAB PAM HPGD GJB2 NR2F2 PTCH1 |
| 0,00025262 | 7 | 72 | Ovulation cycle | PGR EREG ANXA1 BMPR1B PAM SLIT2 SLIT3 |
| 0,00025262 | 16 | 464 | Skin development | ANXA1 SPRR3 SPRR2A TGM1 CSTA SCEL DKK1 ALOX12 PPL KRT17 STMN1 EREG SPINK5 DSG1 KLK13 KRT13 |
| 0,00025262 | 59 | 3779 | Animal organ development | TACC3 ANXA1 SPRR3 SPRR2A TGFBR3 TGM1 DKK1 CSTA SCEL SLIT2 UNC5C DCN PTPRU XRCC1 PGR PCSK5 MTHFD1 CRISPLD2 SFRP4 AGR2 ALOX12 NSD2 CRYAB OAS2 STMN1 PPL EDN3 EREG HIVEP3 KRT17 EPHB2 BRIP1 BMPR1B GCNT3 PML PAM IL18 EDNRA HMGB2 HPGD GJB2 E2F7 AR SLIT3 NR2F2 PTCH1 RELN PCDH18 FAT4 MME GPR68 PTPRM CD24 EPHA3 SPINK5 DSG1 KLK13 CXCL10 KRT13 |
| 0,00025262 | 56 | 3529 | Regulation of molecular function | CX3CL1 SERPINB1 OAS3 OAS2 SASH1 ANXA1 UHRF1 CFTR EPHA3 SFRP4 DKK1 CSTA CDKN2C SLPI EDN3 EREG SORL1 NLRC5 IL18 CSTB RFC4 HMGB2 A2ML1 EPGN NR2F2 CD24 TYMP TGFBR3 ADCYAP1R1 ALOX12 CRYAB ENDOU PPP1R3C EPHB2 SPINK5 IL1RN CXCL9 PML SLIT2 SPINK7 DEPTOR PPP1R9A ALDH1A1 AR CXCL10 RELN SPON1 XRCC1 LAMP3 STMN1 CEP85 PTCH1 H1F0 PCSK5 GRIA2 EDNRA |
| 0,00027708 | 44 | 2498 | Regulation of catalytic activity | CX3CL1 SERPINB1 OAS3 OAS2 SASH1 ANXA1 UHRF1 EPHA3 DKK1 CSTA CDKN2C EDN3 EREG SORL1 NLRC5 IL18 CSTB RFC4 HMGB2 A2ML1 EPGN NR2F2 CD24 ADCYAP1R1 ALOX12 CRYAB PPP1R3C SLPI EPHB2 SPINK5 PML SLIT2 SPINK7 DEPTOR PPP1R9A ALDH1A1 AR CXCL10 RELN XRCC1 LAMP3 CEP85 PCSK5 EDNRA |
| 0,00035135 | 66 | 4507 | Response to stress | CX3CL1 XRCC1 LYZ POLA1 SASH1 PPL MASP1 ANXA1 BRIP1 IL1RN CXCL9 PML NLRC5 HMGB2 KLK7 CXCL10 CIITA YOD1 WDHD1 IL18 CFTR APOL1 LIG1 AGR2 DKK1 OAS3 SLPI CRNN LCN2 E2F7 DPP4 SLC7A2 DCN TGFBR3 ADCYAP1R1 ALOX12 NSD2 CRYAB OAS2 ITGB6 IL1R1 EREG KLK8 SPINK5 SAA2 NT5E BMPR1B PAM MMS22L EDNRA ENPP3 GJB2 UPP1 RELN GPX3 IRF9 UHRF1 CD24 KIAA1324 MMP3 CRISP3 APOB GPR68 NUP210 SPRR3 RFC4 |
| 0,00052113 | 14 | 385 | Response to molecule of bacterial origin | IL1RN CXCL9 CXCL10 CX3CL1 IL18 DCN APOB SLPI LCN2 HMGB2 HPGD GJB2 SASH1 CD24 |
