# Supplementary figures and images for: Markers of Angiogenesis, Lymphangiogenesis, and Epithelial–Mesenchymal Transition (Plasticity) in CIN and Early Invasive Carcinoma of the Cervix: Exploring Putative Molecular Mechanisms Involved in Early Tumor Invasion
Source: Int J Mol Sci. 2020 Sep 6;21(18):6515. doi: 10.3390/ijms21186515 (PMC7554870; doi:10.3390/ijms21186515)

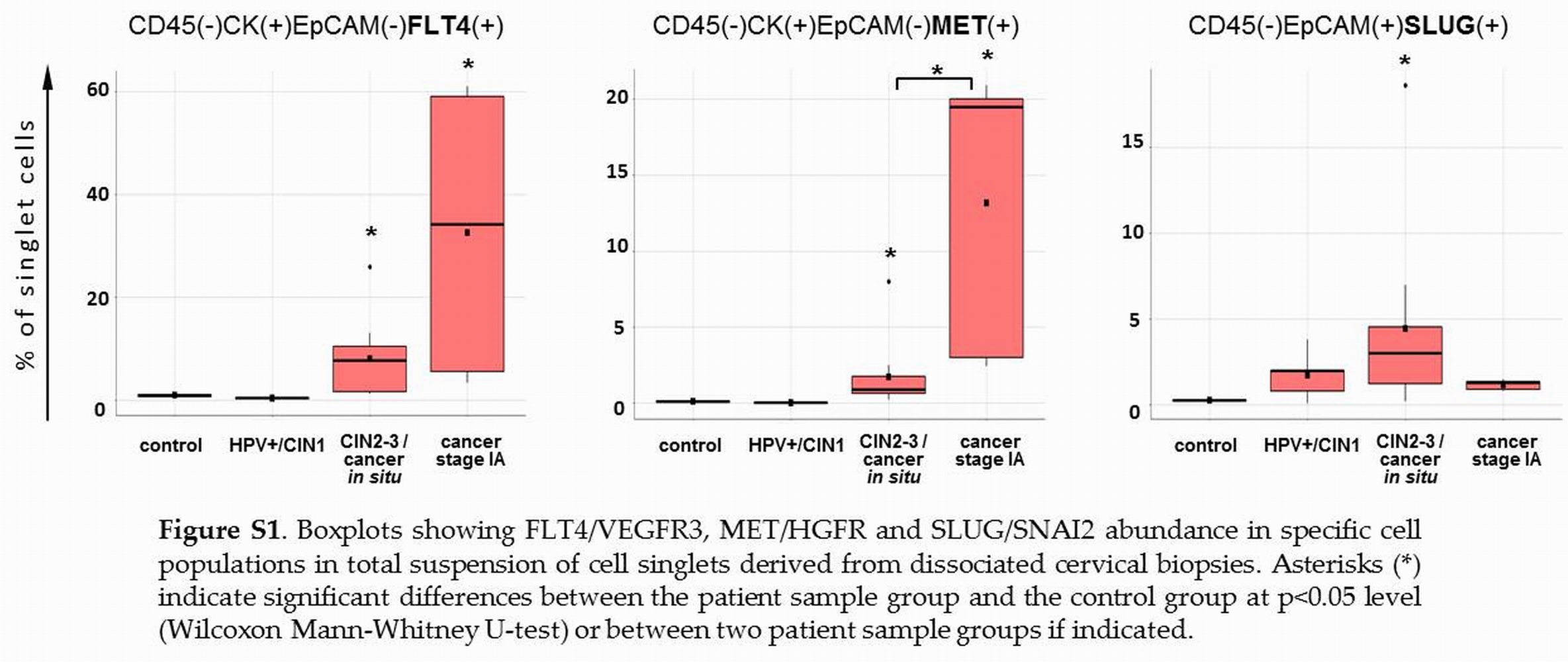

Supplement: Supplementary file 1 [file ijms-21-06515-s001.zip › Supplementary Files - revised/Figure_S1_revised.jpg]

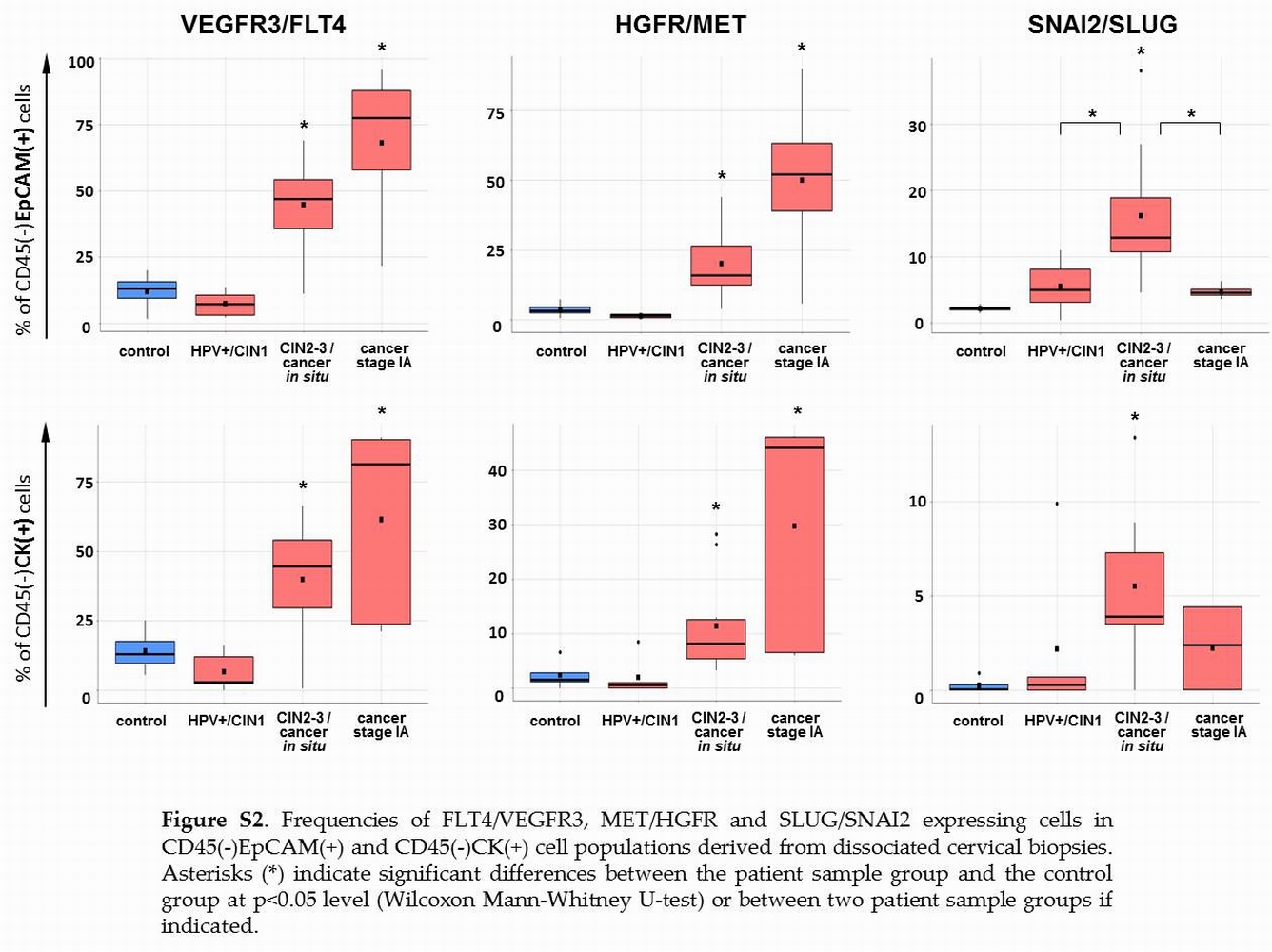

Supplement: Supplementary file 1 [file ijms-21-06515-s001.zip › Supplementary Files - revised/Figure_S2_revised.jpg]
